# Quantifying dexterity and grip strength associations between age, cognitive function (MoCA), and cerebellar gray matter volume in healthy adults across the adult lifespan

**DOI:** 10.1007/s00429-026-03213-6

**Published:** 2026-10-22

**Authors:** Pranesh Rajesh Kannan, Raghav Pallapothu, Santosh Kudaravalli, Sriya Pallapothu, Roger D. Newman-Norlund

**Affiliations:** Department of Psychology, McCausland College of Arts and Sciences, University of South Carolina, Columbia, SC USA

**Keywords:** Cerebellum, Crus II, Grip strength, Manual dexterity, Cognitive aging, Gray matter volume

## Abstract

Cerebellar Crus I/II gray matter volume (GMV) is a structural marker of cognitive aging, and motor performance predicts age-related cognitive decline. Whether grip strength and manual dexterity dissociate in their cerebellar and cognitive correlates is unknown. We computed Pearson partial correlations (age, sex, and race covaried) between grip strength, Nine-Hole Peg Test (9HPT) dexterity, cerebellar Crus I/II GMV, and Montreal Cognitive Assessment (MoCA) subscores in 304 healthy adults aged 20–79 from the University of South Carolina Aging Brain Cohort. GMV was derived from T1-weighted MRI using CAT12 with the AAL3 atlas. After false discovery rate (FDR) correction, dominant 9HPT time correlated with MoCA total score (*r*=-.240) and memory index (*r*=-.220; both pFDR<0.001). Grip strength was unrelated to any MoCA measure but correlated bilaterally with Crus II GMV (*r*=.156–.224, all pFDR≤0.041). Bootstrapped mediation indicated that dexterity statistically accounted for 28.8% of the age–cognition association and Crus II volume for 34.4–41.4% of the age–grip association. Dichotomizing at the MoCA cut-off of 26, each additional second of peg time carried 17.6% higher odds of scoring below cut-off (odds ratio = 1.176, 95% CI [1.062,1.302]) while grip strength carried none. Grip strength, but not dexterity, was independently associated with Crus II GMV, whereas dexterity, but not grip, was associated with cognition. Grip strength and dexterity are therefore not interchangeable indices of motor function. Because the design is cross-sectional, mediation estimates describe shared variance rather than causal pathways, and the pronounced MoCA ceiling likely attenuated the cognitive associations.

**Supplementary Information:**

The online version contains supplementary material available at https://doi.org/10.1007/s00429-026-03213-6.

## Introduction

Alzheimer’s disease (AD) is the leading cause of dementia, accounting for an estimated 60–80% of cases and producing characteristic neuropathological signatures including amyloid-*β* plaques, neurofibrillary tau tangles, synaptic dysfunction, and progressive neurodegeneration (McKhann et al. 2011; Alzheimer’s Association 2023; Jack et al. 2018). Although clinical dementia is most prevalent in older adulthood, the structural and molecular changes that underlie AD emerge decades before the onset of overt cognitive symptoms (Jack et al. 2018; Sperling et al. 2011), motivating a long-standing search for scalable early indicators of brain vulnerability and resilience across the adult lifespan. Established clinical instruments such as the Montreal Cognitive Assessment (MoCA) (Nasreddine et al. 2005) are well validated for detecting overt impairment but are insufficiently sensitive to the subtle cognitive shifts that characterize this preclinical window (Lindvall et al. 2024). Because conventional AD biomarker pipelines based on cerebrospinal fluid, positron emission tomography, and emerging plasma assays remain costly or require specialized infrastructure (Jack et al. 2018), there is increasing interest in functional phenotypes that can index brain integrity inexpensively and at population scale, with motor performance emerging as a particularly promising candidate that explains substantial variance in global cognition independent of demographic and health covariates (Schneider et al. 2025; Jost et al. 2025).

Within this functional-phenotype framework, grip strength has long been the most extensively studied indicator of physical reserve in aging. Hand-grip dynamometry predicts incident frailty, disability, and mortality in older adults (Bohannon 2008, 2019), and longitudinal evidence demonstrates a bidirectional coupling between grip strength and cognitive performance such that stronger grip predicts slower cognitive decline while higher cognition predicts better preserved grip (Cui et al. 2021, 2024). Frailty itself is increasingly conceptualized as a multidimensional syndrome in which sarcopenia, metabolic dysregulation, and cardiovascular processes jointly compromise nervous-system integrity (Jost et al. 2025; Anderer 2025). However, when grip strength is modeled alongside more cognitively demanding motor tasks, fine-motor measures such as the Nine-Hole Peg Test emerge as the dominant independent predictors of global cognition (Schneider et al. 2025), suggesting that more complex motor assessments may better capture the neural processes underlying age-related cognitive decline. Dexterity-based tasks appear particularly sensitive to cognitive load: dual-task paradigms demonstrate that adding a concurrent cognitive demand disproportionately degrades pegboard performance relative to other motor tasks (Petrigna et al. 2020), and visuospatial memory exerts measurable mediating effects on fine motor skill learning in older adults (Hooyman et al. 2023). Together, these findings raise the possibility that grip strength and dexterity, although routinely grouped under “motor function,” capture meaningfully different facets of central nervous system aging.

The cerebellum has emerged over the past three decades as a central structure that participates in both motor control and higher-order cognition. Once viewed as a purely motor structure, the cerebellum is now understood to support a broad range of functions including working memory, executive control, attention, language, and social cognition (Buckner 2013; Stoodley and Schmahmann 2009). This functional breadth reflects an anatomical division of labor within the cerebellum itself: anterior cerebellar lobules participate primarily in sensorimotor processing, whereas the posterior cerebellum, particularly the lateral hemispheres encompassing Crus I and Crus II, supports higher-order cognitive operations (Stoodley et al. 2012; Stoodley and Schmahmann 2009). The cerebellum is also among the structures most affected by normative aging, with substantial volumetric change observed across the adult lifespan (Ramanoël et al. 2018). Within this organization, grip strength and fine motor dexterity engage cerebellar circuitry in dissociable ways. Grip strength is determined by maximum voluntary force production and reflects a combination of central neural drive and substantial peripheral contributions including forearm musculature, neuromuscular junction integrity, and overall muscle mass (Bohannon 2019; Ward et al. 2007). In contrast, fine motor dexterity, as indexed by the Nine-Hole Peg Test, depends on the cerebellum’s contribution to error-based learning, online movement correction, and the temporal coordination of sequential finger movements (Lemon 2008; Stoodley et al. 2012). Because much of the existing literature has treated grip strength as a general proxy for neurological health without explicitly accounting for these peripheral contributions (Bohannon 2019), the more specific cerebellum–behavior associations afforded by fine motor measures may have been systematically obscured.

Among cerebellar regions, Crus I and Crus II warrant a particular a priori focus in the context of cognitive aging. As the supramodal zone of the posterior cerebellar hemisphere, Crus I and Crus II are anatomically and functionally distinguished from primary sensorimotor cerebellum (Buckner 2013; Stoodley et al. 2012; Stoodley and Schmahmann 2009). Structural aging studies have identified the right cerebellar Crus I/II region as showing reduced gray matter volume in older adults relative to younger comparison groups (Ramanoël et al. 2018), supporting its sensitivity to normative neural aging. Across the AD spectrum, voxel-based morphometry has revealed that Crus I atrophy emerges selectively at the AD dementia stage (Toniolo et al. 2018), while Crus II atrophy is detectable across the continuum from subjective cognitive decline through mild cognitive impairment (MCI) to AD dementia and tracks neuropsychological performance (Van Rossem et al. 2025). Cerebellar functional connectivity work converges on these regions, with progressive reductions in Crus I connectivity from MCI to AD tracking memory, executive function, visuospatial ability, and global cognition (Zhou et al. 2021), and disrupted Crus II–thalamic coupling linked to attention deficits in MCI and broader cognitive decline in AD (Tang et al. 2021). Causal animal work further confirms that Crus II neurons directly modulate recognition and spatial memory via a polysynaptic pathway through the cerebellar lateral nucleus and ventromedial thalamus (Fan et al. 2025). Together, this convergent structural, functional, and causal evidence positions Crus I and Crus II as cerebellar nodes that are particularly vulnerable in cognitive aging and well suited to interrogating brain–behavior relationships in mixed motor–cognitive constructs.

The present study therefore examined associations between grip strength, Nine-Hole Peg Test dexterity, cerebellar Crus I/II gray matter volume, and domain-specific cognitive performance assessed by the MoCA in a cross-sectional lifespan sample of healthy adults aged 20–79. We tested whether grip strength and dexterity dissociate in their cerebellar structural correlates and in their relationships to global cognition and individual MoCA subdomains, and used bootstrapped mediation analyses to evaluate whether dexterity and Crus II volume statistically account for portions of age-related variance in cognition and grip strength, respectively. By distinguishing the cerebellar and cognitive signatures of gross and fine motor performance, this work aims to clarify which motor measures are most informative for indexing cerebellar integrity in normative cognitive aging.

## Materials and methods

### Participants

Data from the NIH Toolbox physiological measures were drawn from the University of South Carolina’s Aging Brain Cohort Study Repository, a multimodal lifespan database for studying the relationship between the brain, cognition, genetics, and behavior in healthy aging. As part of this study, T1-weighted magnetic resonance imaging (MRI) images were collected, and participants also completed grip strength and dexterity testing. Grip strength was tested using a Jamar Plus Dynamometer, with force recorded in pounds (lb), and dexterity was tested using the Nine-Hole Peg Test for both dominant and non-dominant hands. Grip strength and dexterity assessments were administered in accordance with standardized NIH Toolbox motor function protocols (National Institutes of Health Toolbox 2012). Data were collected between September 2019 and December 2023. For every participant, the cognitive battery (including the MoCA), grip strength, and dexterity assessments were completed within 48 h of the MRI scan, so the measures entered into the analyses reported here are effectively contemporaneous. Health-related questionnaires capturing demographic information were in some cases administered earlier in the enrollment process; all study procedures were typically completed within a three-week window. Scanner and sequence details are given in Scanning Protocol, below.

The University of South Carolina Institutional Review Board (IRB) approved this procedure. The 304 participants were between 20 and 79 years old (mean age = 46.74 years, SD = 19.38; 75 males, 229 females). By race, the sample comprised 251 White (82.6%), 32 Black or African American (10.5%), 12 Asian (3.9%), 8 Unknown or not reported (2.6%), and 1 participant reporting more than one race (0.3%). Eligibility for MRI scanning required participants to tolerate lying supine for approximately one hour, have a maximum girth below 60 inches, and weigh under 400 pounds. Participants did not have existing medical conditions including, but not limited to: severe illnesses such as cancer, untreated and unmanaged psychological conditions such as schizophrenia, and no current or past fatiguing illnesses. The sample comprised all participants in the Aging Brain Cohort repository meeting inclusion criteria for whom MoCA and neuroimaging data were available. Grip strength or dexterity was missing for a small number of these participants, and analyses were therefore conducted on complete cases within each model (see Statistical Methods). This dataset specified the sex, age, socioeconomic status, and race of each individual as fixed variables. Two additional individuals whose recorded sex was neither male nor female were not retained, because sex was entered as a covariate in every model and no stratified or moderation estimate could be obtained for a group of that size; the final analytic sample was therefore 304. We note at the outset that the sex distribution is markedly unbalanced (75 men, 229 women); the consequences of this imbalance for the grip strength analyses are addressed directly in Sensitivity Analyses and in the Discussion.

### Scanning protocol

All participants underwent the same MRI scanning protocol at the McCausland Center for Brain Imaging (MCBI), located at Prisma Health Hospital, Columbia, South Carolina, on a Siemens 3 T Prisma Fit scanner equipped with a 20-channel head coil. T1-weighted images were used for volumetric analyses and were acquired using the following parameters: T1-weighted magnetization-prepared rapid gradient-echo (MP-RAGE) sequence with 1 mm isotropic voxels, 256 × 256 matrix size, 9° flip angle, and 192-slice sequence with repetition time = 2250 ms, inversion time = 925 ms, and echo time = 4.11 ms.

### Cognitive assessment

Global cognition was assessed with the Montreal Cognitive Assessment (MoCA), administered and scored by trained study personnel following the standard manual (Nasreddine et al. 2005). In addition to the total score (0–30), the seven MoCA domain index scores (memory, executive function, attention/concentration, language, visuospatial, orientation, and immediate recall) were derived from item-level responses and analyzed as separate domain outcomes. The MoCA was administered within 48 h of the motor assessments and the MRI scan.

The MoCA was selected with full awareness of its limitations in a cognitively healthy lifespan sample. It was designed as a screening instrument for mild cognitive impairment and is known to exhibit a ceiling effect and limited sensitivity to preclinical change in unimpaired adults (Lindvall et al. 2024); the magnitude of that ceiling in the present sample is quantified explicitly in the Results. Three considerations nonetheless motivated its use. First, the aim of this study was not to detect preclinical Alzheimer’s disease but to ask whether two routinely collected motor measures differ in the degree to which they track a standard, clinically interpretable index of cognitive status; the ubiquity of the MoCA in clinical and research settings is precisely what makes a demonstrated association with it translationally useful. Second, the MoCA yields domain index scores, which allowed us to test whether any motor–cognition association is domain-selective rather than global, a question a single composite score cannot address, and one central to the dissociation tested here. Third, a ceiling effect generally acts as a conservative bias: restriction of range at the upper end of the scale tends to attenuate true associations toward zero, so an association that survives it is unlikely to be inflated by it. Because attenuation is nonetheless a real threat to the null result for grip strength, cognition is additionally analyzed with Spearman rank correlations of the covariate-adjusted residuals and with the sample dichotomized at the conventional MoCA cut-off of 26, the latter of which does not rely on variation above the cut-off.

### Gray matter volume extraction

**Fig. 1:**
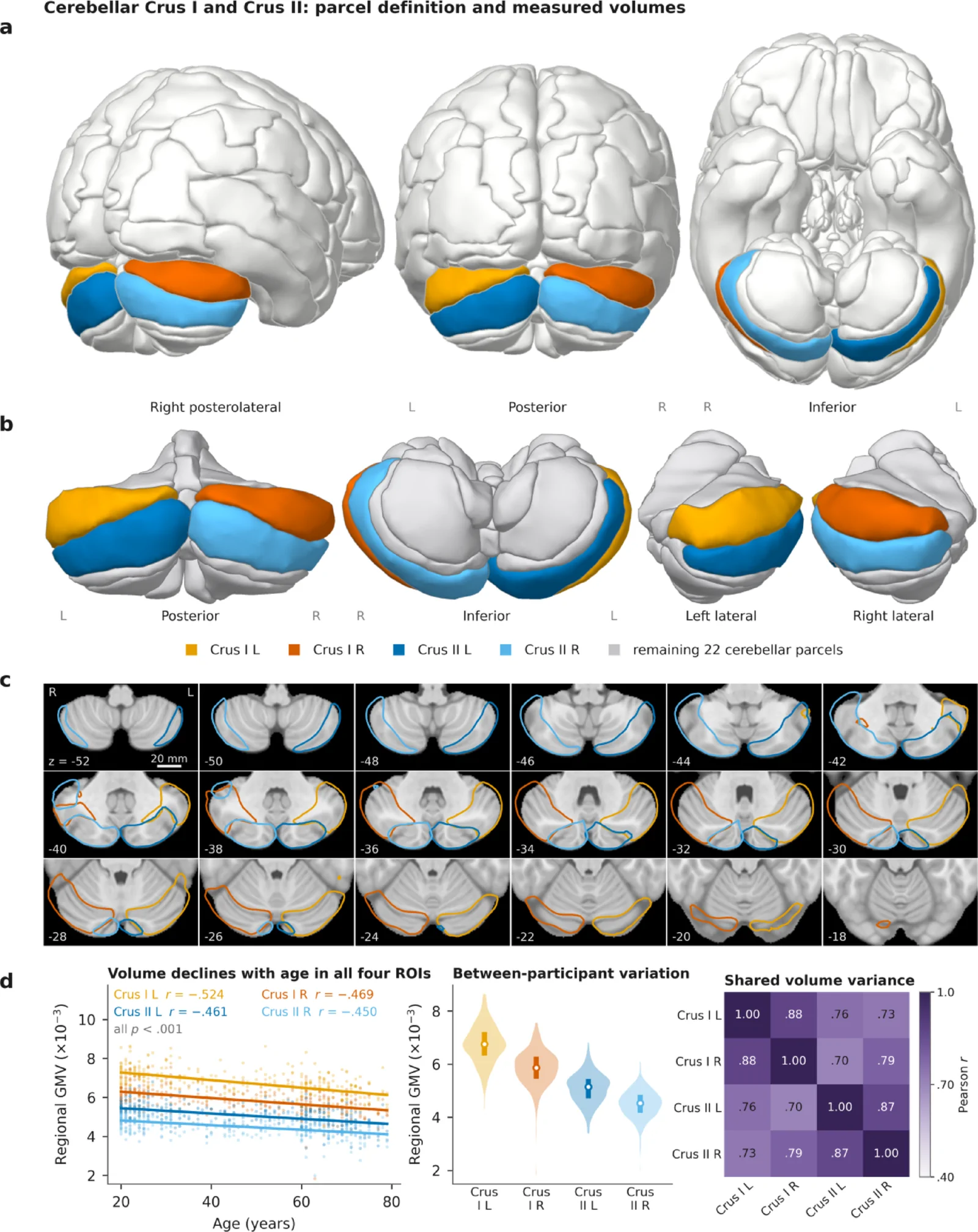
Cerebellar Crus I and Crus II: parcel definition and measured volumes. **a** The four a priori regions of interest, left and right Crus I and Crus II, shown on a whole-brain surface in right posterolateral, posterior, and inferior views. **b** The same four parcels on the isolated cerebellum in posterior, inferior, left lateral, and right lateral views; the remaining 22 cerebellar parcels of the AAL3 atlas are shown in gray. Hemisphere is marked on each view: posterior views place the left hemisphere on the image left, and inferior views place it on the image right. **c** Axial slices through the cerebellum from z = − 52 to − 18 mm in 2-mm steps, with each region of interest outlined on the MNI152 template. **d** Left: regional gray matter volume as a function of age, with least-squares fits; volume declines with age in all four regions (all *p*<.001). Center: between-participant distributions of each regional volume. Right: Pearson correlations among the four regional volumes. Surface and slice renderings were produced with brainWhiz (Newman-Norlund 2026); parcels are from the AAL3 atlas (Rolls et al. 2020; Tzourio-Mazoyer et al. 2002)

To extract regional gray matter volumes, the Computational Anatomy Toolbox (CAT12) was employed with default settings (Gaser et al. 2024). CAT12 for voxel-based morphometry (VBM) employs a fully automated workflow that quantifies brain structure from MRI scans. The pipeline applies bias correction to minimize MRI artifacts, segments the brain into gray matter, white matter, and cerebrospinal fluid via tissue classification, spatially normalizes the images to a Montreal Neurological Institute (MNI) template, corrects for local brain size, and modulates the segments to preserve volume information. Cerebellar regions of interest (ROI) were defined using the Automated Anatomical Labeling 3 (AAL3) atlas (Rolls et al. 2020; Tzourio-Mazoyer et al. 2002) as bundled within the CAT12 pipeline. Four a priori regions of interest were extracted: left Crus I, right Crus I, left Crus II, and right Crus II. All four were treated as separate unilateral volumes at every stage of analysis; left and right volumes were never summed, averaged, or otherwise combined into a single bilateral measure, and each entered the FDR family as its own test. Where a result is subsequently described as “bilateral,” this denotes that the left and the right region of interest of that lobule each showed the effect independently in its own test, not that the hemispheres were pooled. To control for individual differences in head size, regional volumes were expressed as a proportion of each participant’s total intracranial volume (TIV). The four ROIs are shown in Fig. 1, both as surface renderings and as outlines on the MNI152 template, together with the distribution of the measured volumes and their decline with age.

### Statistical methods

All statistical analyses were conducted in Python (version 3.13) using the pandas, NumPy, SciPy, and statsmodels libraries. Statistical significance was set at α = 0.05. Associations between variables were assessed using Pearson partial correlations, computed via an ordinary least squares (OLS) residualization approach: each variable of interest was regressed on the covariate set using OLS, and the Pearson correlation was then computed between the resulting residuals. Significance was evaluated on *n* − 2 − k degrees of freedom, where k is the number of covariates, so that the covariates consumed in the residualization step are not counted as free observations. Age was entered as a continuous covariate. Sex and race were treated as categorical variables and dummy-coded prior to entry, with male sex and Asian race serving as the omitted reference categories, yielding four dummy-coded race regressors and one dummy-coded sex regressor. The choice of omitted level does not affect the partial correlations reported here, because the four race dummies span the same column space whichever level is dropped. This approach was used in preference to treating sex and race as continuous numeric codes, which would impose an unwarranted ordinal structure on nominal variables. Renderings of cerebellar anatomy in the figures were produced with brainWhiz (Newman-Norlund 2026), an interactive viewer for multi-atlas parcellations and region-mapped data.

False discovery rate (FDR) correction was applied using the Benjamini–Hochberg procedure. For Crus I/II (L and R) × motor measure associations (dominant and non-dominant, grip strength and dexterity), correction was applied across the full family of 16 tests (four ROIs × four motor outcomes). For dexterity × MoCA associations, correction was applied across eight tests (dominant dexterity × MoCA total score and seven index subscores). Non-dominant dexterity and grip strength × MoCA associations were examined separately and evaluated at uncorrected *α* = 0.05 given their exploratory nature; no associations in these families survived even uncorrected thresholds. Two-tailed *p*-values were used for all FDR corrections.

A further set of partial correlations was computed after the analyses described above had been completed, and is reported throughout as post hoc and exploratory. It was not pre-specified, and it does not contribute to the confirmatory claims of this paper. Partial correlations between regional gray matter volume and each motor and cognitive measure were computed across the 26 cerebellar parcels of the AAL3 atlas, with Benjamini–Hochberg correction applied across those 26 parcels within each measure. This family is additional to, and independent of, the 16-test and eight-test families described above. Because the Benjamini–Hochberg procedure depends on the composition of the family to which it is applied, an association may survive correction in one family and not in another; where they disagree, the pre-specified families take precedence. The analysis was undertaken to place the a priori findings in anatomical context, showing where within the cerebellum the observed associations fall relative to every other parcel, rather than to test any additional hypothesis.

Bootstrapped mediation analyses were conducted to test indirect effects of age on grip strength (via Crus II gray matter volume) and on MoCA total score (via dominant hand dexterity). For each model, age served as the independent variable (*X*), the proposed mediator (*M*) was either Crus II volume or dexterity, and the dependent variable (*Y*) was either grip strength or MoCA total score. Sex and race, coded nominally, were entered as covariates in all paths. Path coefficients were estimated using OLS regression. Indirect effects (*a*×*b*) were computed as the product of the path *a* coefficient (*X*→*M*, controlling for covariates) and the path *b* coefficient (*M*→*Y*, controlling for *X* and covariates). Statistical significance of indirect effects was evaluated using 5,000 bootstrap iterations with percentile 95% confidence intervals (CI); an indirect effect was considered significant if the confidence interval excluded zero. The proportion of the total effect mediated was computed as the indirect effect divided by the total effect (*c* path).

Because the present design is cross-sectional, these mediation models are interpreted strictly as a statistical decomposition of covariance and not as evidence of a causal pathway. Independent variable, mediator, and outcome were all measured at the same time point, so the temporal precedence required for causal mediation is not established, and the same covariance structure is equally compatible with alternative orderings: reverse causation, or a common third cause such as global neurodegeneration, cardiovascular burden, or physical activity acting on cerebellar volume, grip strength, and cognition together. We therefore report indirect effects and the proportion of the total effect mediated as descriptive quantities, use the term “mediation” in its statistical sense throughout, and avoid causal verbs when describing these results. Longitudinal data are required to test the causal hypotheses that these models are intended to generate.

To formally evaluate the proposed double dissociation, the strengths of competing overlapping partial correlations were compared using the procedure of Meng et al. (1992) for dependent correlations that share a common variable. Two planned contrasts were tested: whether left Crus II volume was more strongly associated with grip strength than with dexterity, and whether MoCA total score was more strongly associated with dexterity than with grip strength. For these comparisons, motor measures were sign-aligned so that higher values denoted better performance, and age, sex, and race were partialled from all variables before comparison; two-tailed p-values are reported.

Three sets of sensitivity analyses were added in revision. First, because women outnumbered men by roughly three to one, the two focal associations (Crus II volume with grip strength; dominant dexterity with MoCA total score) were re-estimated separately within each sex, controlling for age and race, compared across sexes using Fisher’s r-to-z test for independent correlations, and tested formally with a Crus II × sex product term in an ordinary least squares model predicting grip strength (both raw and with all continuous variables standardized). The primary Crus II × grip family was additionally re-estimated within the female subsample alone. Second, because MoCA scores were compressed against the instrument ceiling, the dexterity–cognition associations were re-estimated as Spearman rank correlations of the covariate-adjusted residuals, and the sample was dichotomized at the conventional MoCA cut-off (< 26 versus ≥ 26) and analyzed with logistic regression predicting below-cut-off status from dominant dexterity and dominant grip strength entered simultaneously, controlling for age, sex, and race. Third, the sample was split at 50 years into younger (20–49) and older (50–79) strata and the focal associations re-estimated within each, again compared using Fisher’s r-to-z. These analyses are reported as sensitivity and generalizability checks rather than as new confirmatory tests; they were not FDR-corrected and are interpreted qualitatively, in terms of whether the primary effects hold within subgroups and under assumptions that the primary analyses do not make.

Analyses were conducted on all available complete cases for each specific analysis, with listwise deletion applied per model. Sample sizes ranged from 297 to 304 across analyses, reflecting missing values in grip strength (*n* = 5–6 missing) and dexterity (*n* = 6–7 missing) assessments. With a sample of approximately 300 participants, the present study had approximately 80% power to detect partial correlations of *r*≥.16 (two-tailed, *α* = 0.05).

Large language model tools were used during the preparation of this work. Their use extended beyond copyediting: they assisted in drafting and debugging the Python analysis scripts, and in locating candidate literature during the writing of the Introduction and Discussion. All analyses were specified by the authors, all code was reviewed and executed by the authors, and all statistical output reported here was verified against the underlying data by the authors, who take full responsibility for the content of this manuscript. No text was generated and used without author revision, and no tool was used to interpret results or to draw scientific conclusions.

## Results

The final analytic sample comprised 304 participants (75 male, 229 female; mean age = 46.74 ± 19.38 years, range: 20–79). Descriptive statistics for all primary variables are presented in Table 1.

**Table 1:** Descriptive statistics for demographic, motor, cognitive, and cerebellar volume measures

Variable	*N*	Mean	SD	Min	Max
Age (years)	304	46.74	19.38	20.00	79.00
Grip strength − dominant (lb)	299	71.83	23.07	15.40	156.90
Grip strength − non-dominant (lb)	298	68.03	23.87	17.69	161.80
Dexterity − dominant (s)	298	19.24	3.56	12.55	37.66
Dexterity − non-dominant (s)	297	20.07	3.12	12.48	31.78
MoCA total	304	27.33	2.40	15.00	30.00
Crus I − left	304	0.0068	0.0007	0.0043	0.0086
Crus I − right	304	0.0059	0.0007	0.0018	0.0076
Crus II − left	304	0.0051	0.0006	0.0034	0.0068
Crus II − right	304	0.0045	0.0005	0.0020	0.0061

Sample size (N), mean, standard deviation (SD), minimum, and maximum for each measure (*N* = 304). Grip strength is in pounds (lb); dexterity is Nine-Hole Peg Test completion time in seconds (s). Cerebellar Crus I and Crus II gray matter volumes (left and right) are expressed as a proportion of total intracranial volume. N varies across measures owing to missing grip strength and dexterity assessments. Consistent with expectations for a cognitively healthy lifespan sample, MoCA total scores were strongly negatively skewed and compressed against the instrument’s ceiling (mean = 27.33, SD = 2.40, median = 28, observed range 15–30, skewness=-1.27). Fifty-nine participants (19.4%) obtained the maximum score of 30, 169 (55.6%) scored 28 or above, and 238 (78.3%) scored at or above the conventional cut-off of 26; the observed standard deviation spanned only 16.0% of the observed range. The MoCA memory index was more compressed still, with 53.9% of participants at the maximum of 15 (skewness=-2.17). Restriction of range of this magnitude attenuates any true association between motor performance and cognition, so the cognitive associations reported below are likely attenuated, and the null result for grip strength should be read with corresponding caution. Analyses that address the ceiling directly are reported under sensitivity analyses

**Fig. 2:**
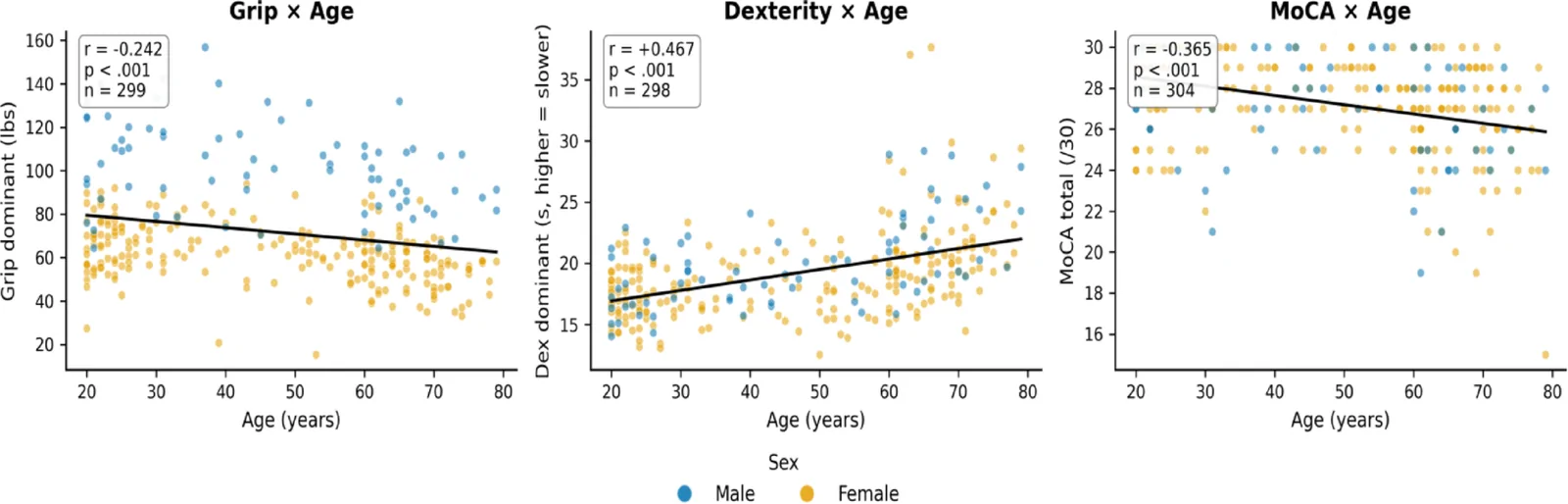
Grip strength, dexterity, and cognition as a function of age. Scatterplots of dominant grip strength (lb), dominant Nine-Hole Peg Test time (s; higher = slower), and MoCA total score against age, with points colored by sex (analytic sample, *N* = 304). Bivariate Pearson correlations, p-values, and n are inset. Grip strength declines and peg time increases with age, whereas MoCA scores are comparatively stable

**Fig. 3:**
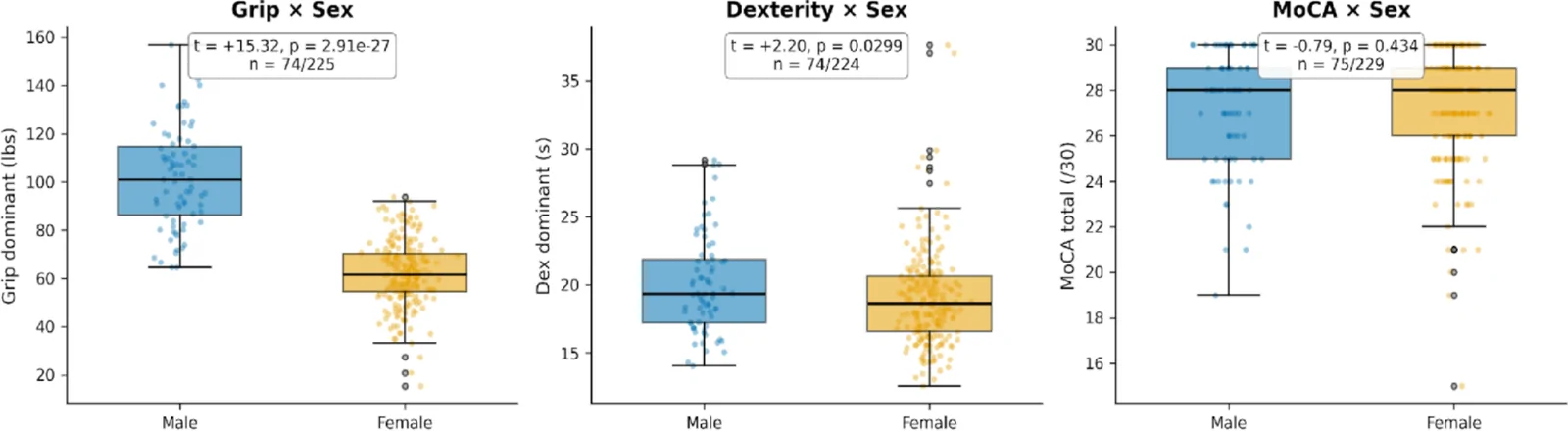
Distributions of grip strength, dexterity, and cognition by sex. Box-and-jitter plots of dominant grip strength (lb), dominant peg time (s), and MoCA total score for male and female participants. Independent-samples t statistics, p-values, and group sizes are inset. Grip strength differs markedly by sex; dexterity differs modestly; MoCA does not

The bivariate distributions of grip strength, dexterity, and MoCA across age and sex are shown in Figs. 2 and 3. Grip strength and dexterity both varied with age, and grip strength differed markedly by sex, whereas MoCA scores were comparatively stable across demographic groups. The corresponding distributions by race are provided in Online Resource 1 (Fig. S1); because race was dummy-coded and entered as a covariate in every inferential model, and because gray matter volume is not reported by race, these panels are descriptive only and support no inferential comparison.

### Relationships between grip strength and dexterity

First, the study sought to understand the relationship between grip strength and dexterity. All four grip strength × dexterity pairings across dominant and non-dominant hands were significantly negatively correlated after controlling for age, sex, and race (dominant grip × dominant dexterity: *r*=-.241, *p*<.001; dominant grip × non-dominant dexterity: *r*=-.154, *p*=.009; non-dominant grip × dominant dexterity: *r*=-.154, *p*=.009; non-dominant grip × non-dominant dexterity: *r*=-.168, *p*=.004; all *n* = 296–299). Higher grip strength was consistently associated with faster dexterity, confirming that the two measures are related but not redundant motor constructs.

### Cerebellar Crus I/II volumes and grip strength

Having established that grip strength and dexterity represent related but distinct motor constructs, the next aim was to determine whether variation in these behaviors corresponded to gray matter volume differences within a priori cerebellar regions of interest. Partial correlations between cerebellar Crus I and Crus II gray matter volumes and grip strength were computed with age, sex, and race entered as covariates. FDR correction was applied across all 16 Crus I/II × motor measure associations.

Cerebellar Crus II volume was positively associated with grip strength following FDR correction. Crus II left correlated significantly with non-dominant grip strength (*r*=.224, pFDR=0.002, *n* = 298) and dominant grip strength (*r*=.156, pFDR=0.041, *n* = 299). Crus II right correlated significantly with non-dominant grip strength (*r*=.198, pFDR=0.005, *n* = 298). In all cases, larger Crus II volume was associated with stronger grip. Cerebellar Crus I showed numerical trends in the same direction for non-dominant grip (Crus I left: *r*=.140, pFDR=0.067; Crus I right: *r*=.131, pFDR=0.081) that did not survive FDR correction. Full results are presented in Table 2.

**Table 2:** Partial correlations between cerebellar Crus I/II gray matter volume and grip strength

ROI	Outcome	*r*	*p* (uncorr.)	*p* (FDR)	*n*
Crus II − left	Grip non-dominant	0.224	< 0.001	0.002*	298
Crus II − right	Grip non-dominant	0.198	< 0.001	0.005*	298
Crus II − left	Grip dominant	0.156	0.008	0.041*	299
Crus I − left	Grip non-dominant	0.140	0.017	0.067	298
Crus I − right	Grip non-dominant	0.131	0.025	0.081	298
Crus II − right	Grip dominant	0.120	0.040	0.107	299
Crus I − left	Grip dominant	0.065	0.271	0.481	299
Crus I − right	Grip dominant	0.060	0.307	0.491	299

Partial correlation coefficient (r), uncorrected p-value, Benjamini–Hochberg FDR-corrected p-value, and sample size (n) for each cerebellar region of interest by grip strength outcome, controlling for age, sex, and race. Rows are ordered by effect size. Significance is evaluated on *n* − 2 - k degrees of freedom, where k is the number of covariates. FDR correction was applied across the full family of 16 Crus I/II by motor associations. Asterisks (*) denote associations surviving FDR correction (FDR-corrected *p* < .05)

**Fig. 4:**
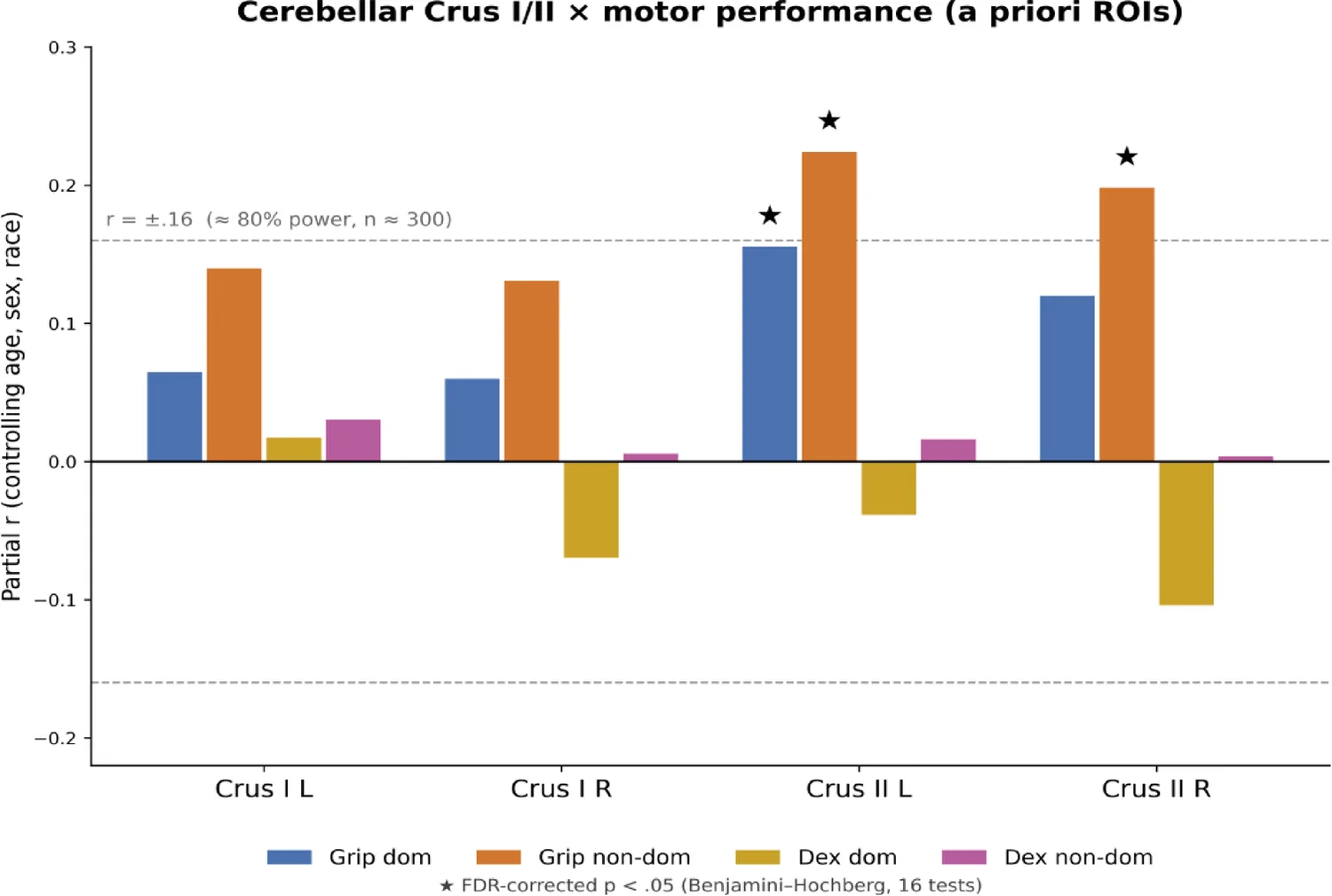
Partial correlations between cerebellar Crus I/II volume and motor performance. Partial correlations (controlling for age, sex, and race) between left and right Crus I and Crus II gray matter volume and four motor outcomes: dominant and non-dominant grip strength and dominant and non-dominant dexterity. The dashed reference line marks r = ± 0.16 (≈ 80% power at *n* ≈ 300). Asterisks mark associations surviving Benjamini–Hochberg FDR correction across the 16-test family (* FDR-corrected *p* < .05)

**Fig. 5:**
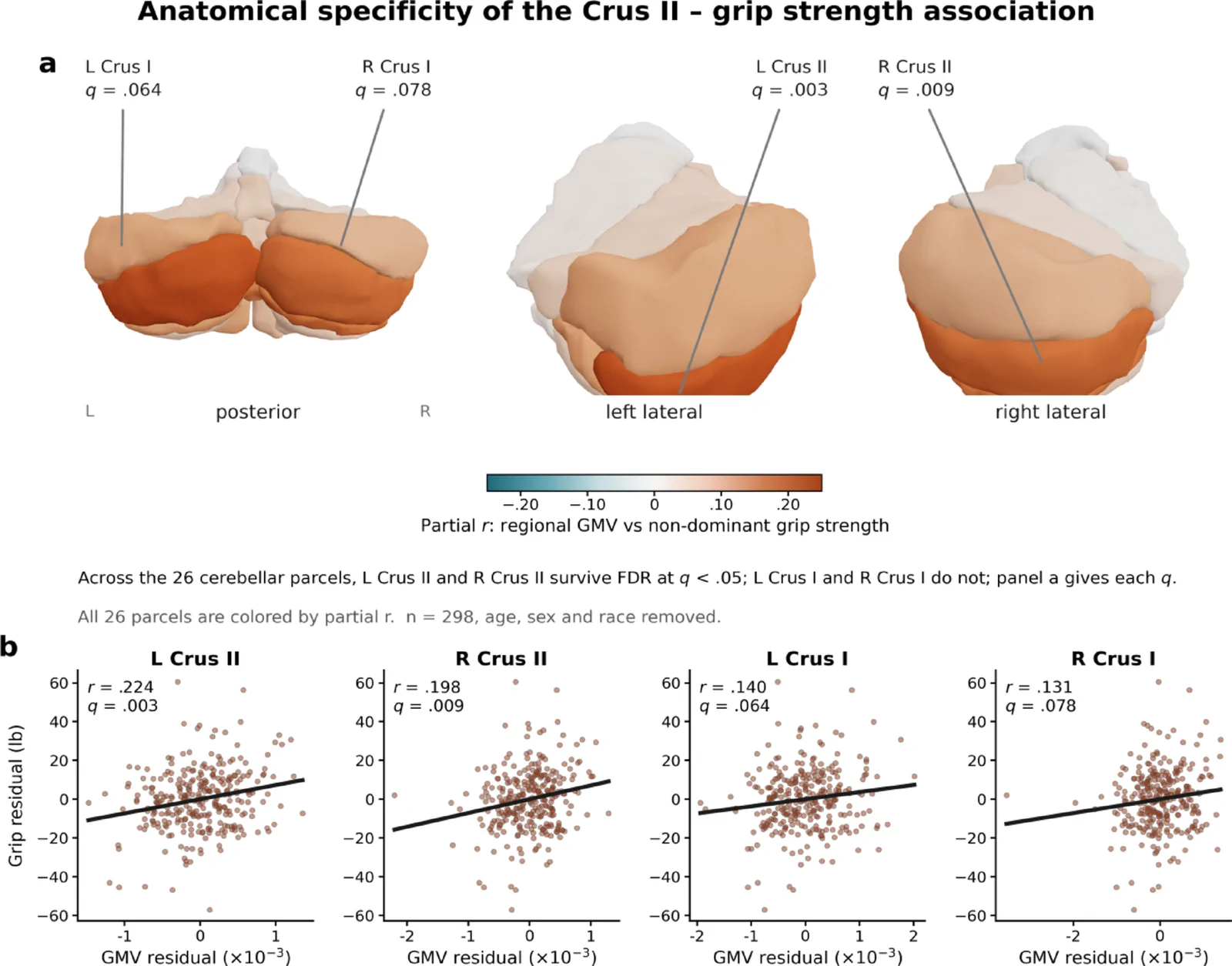
Anatomical specificity of the Crus II–grip strength association. **a** All 26 cerebellar parcels of the AAL3 atlas, colored by their partial correlation with non-dominant grip strength after partialling age, sex, and race, in posterior, left lateral, and right lateral views; the posterior view places the left hemisphere on the image left. Labels mark the four a priori regions of interest with their FDR-corrected p-values across the 26-parcel family. **b** Residual scatterplots of non-dominant grip strength against regional gray matter volume for each of the four a priori regions of interest, after partialling age, sex, and race; partial r and the FDR-corrected p-value across the 26-parcel family are inset. The q values shown in both panels come from the 26-parcel family, which was computed post hoc and is separate from the pre-specified 16-test Crus I/II × motor family reported in Table 2 and Fig. 4 (see Statistical Methods)

The full set of Crus I/II × motor partial correlations is displayed in Fig. 4. Figure 5 shows the anatomical specificity of the Crus II–grip association, with residual scatterplots for each of the four a priori regions of interest in Fig. 5b.

A post hoc exploratory analysis placed these findings in anatomical context (Fig. 5a; Online Resource 1, Fig. S2). With correction applied across the 26 cerebellar parcels, four parcels survived: left Crus II (*r*=.224, q=0.003), right Crus II (*r*=.198, q=0.009), right lobule VIIb (*r*=.190, q=0.009), and vermis VIII (*r*=.174, q=0.019). Lobule VIIb and vermis VIII form part of the second sensorimotor representation of the cerebellum, and their appearance alongside Crus II is consistent with a sensorimotor rather than a cognitive locus for the grip association. Neither Crus I region survived correction within this family (left *r*=.140, q=0.064; right *r*=.131, q=0.078).

Because these exploratory families differ in composition from the pre-specified families, the two can disagree, and one such disagreement should be noted explicitly. The association between left Crus II volume and dominant grip strength survives FDR correction across the pre-specified 16-test family (Table 2; *r*=.156, pFDR=0.041) but not across the 26 cerebellar parcels, where it is evaluated against a larger and differently composed set of tests. Both statements are correct as reported; they answer different questions, and the pre-specified family remains the basis for the confirmatory claims made here.

### Cerebellar Crus I/II volumes and dexterity

After comparing grip strength correlations to selected ROI GMV, dexterity was then correlated with the same ROIs. No significant associations were observed between cerebellar Crus I or Crus II volumes and Nine-Hole Peg Test dexterity performance after FDR correction (all pFDR>0.17). The largest observed uncorrected association was *r*=-.10 (Crus II right × dominant dexterity, *p*=.076, pFDR=0.174). With the present sample size (*n* ≈ 300), the study was adequately powered at approximately 80% to detect correlations of *r*≥.16 (two-tailed, α = 0.05), indicating that these results constitute evidence against moderate-to-large associations rather than a failure of statistical power.

### Dexterity, grip strength, and cognitive performance

**Fig. 6:**
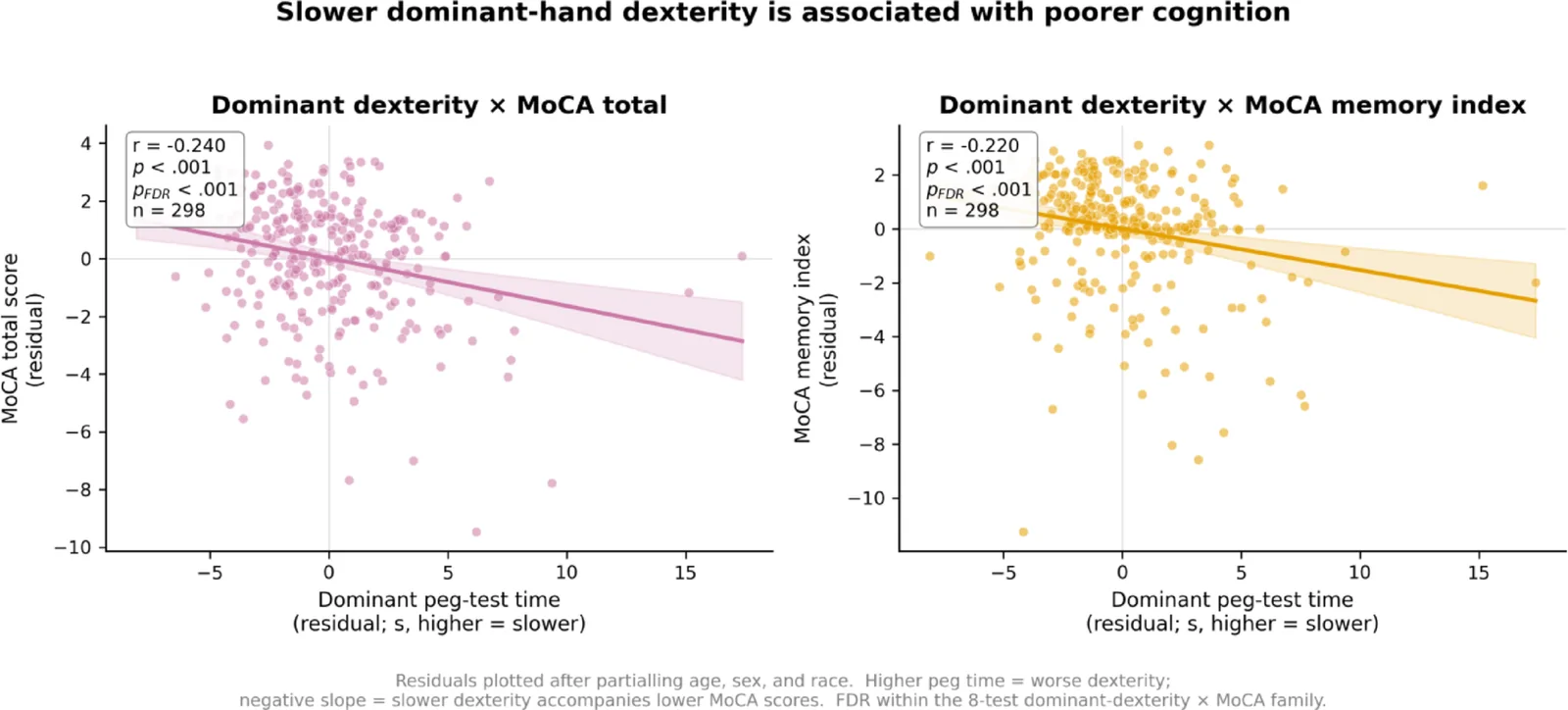
Slower dominant-hand dexterity is associated with poorer cognition. Residual scatterplots of MoCA total score (left) and MoCA memory index (right) against dominant Nine-Hole Peg Test time, after partialling age, sex, and race. Higher peg time indicates slower (worse) dexterity. Partial r, uncorrected and FDR-corrected p-values, and n are inset; shaded bands are 95% confidence intervals. FDR was applied across the eight-test dexterity – MoCA family (Benjamini–Hochberg)

To further evaluate the clinical relevance of motor performance, the next study aim was to determine whether grip strength and dexterity were viable markers of cognition. Partial correlations between motor measures and MoCA total/subscores (memory, executive, visuospatial, orientation, language, attention/concentration, and immediate recall) were conducted controlling for age, sex, and race, with FDR correction applied across eight dexterity × MoCA comparisons. Dominant hand dexterity was significantly associated with MoCA total score (*r*=-.240, pFDR<0.001, *n* = 298) and the MoCA memory index (*r*=-.220, pFDR<0.001, *n* = 298; slower peg time = lower MoCA performance). No other MoCA subscores survived FDR correction (executive index: *r*=-.110, pFDR=0.164; all others pFDR>0.17). Non-dominant dexterity was not significantly associated with MoCA total score or any MoCA subscore at the uncorrected threshold at which this exploratory family was evaluated. Grip strength, whether dominant or non-dominant, was not significantly associated with any MoCA subscore at the uncorrected threshold at which this exploratory family was evaluated (all puncorr>0.07). These dexterity–cognition associations are shown in Fig. 6.

### Cross-sectional mediation: age, dexterity, and cognition

**Fig. 7:**
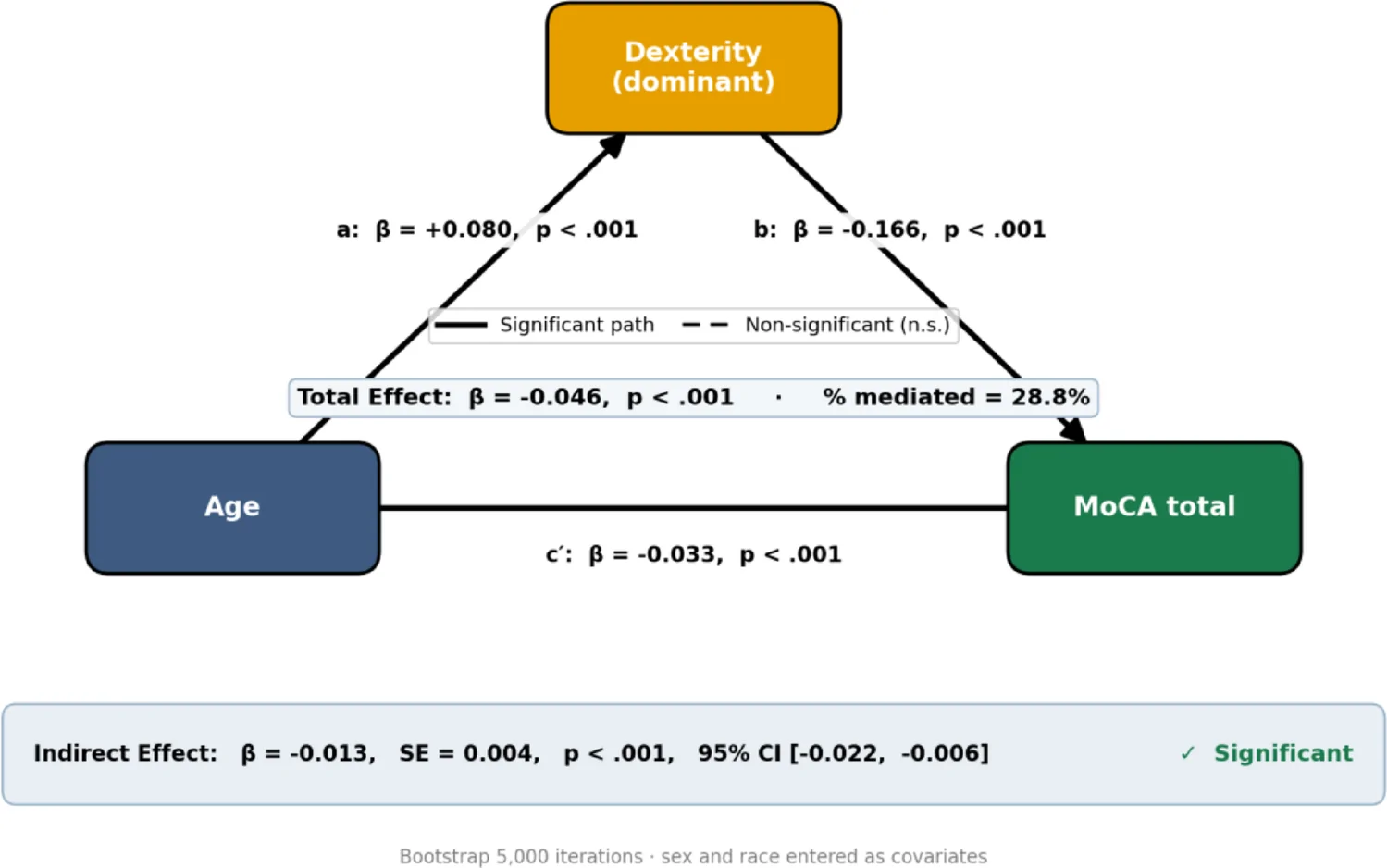
Cross-sectional mediation model relating age, dominant-hand dexterity, and cognition. Mediation model with age as the independent variable, dominant Nine-Hole Peg Test dexterity as the mediator, and MoCA total score as the outcome; sex and race entered as covariates. Path coefficients (**a**, **b**, **c**, **c′**) with standard errors and p-values are shown, together with the bootstrapped indirect effect (5,000 iterations, 95% CI) and the proportion of the total effect mediated. Solid arrows denote significant paths. Paths are estimated from cross-sectional data and describe a statistical decomposition of covariance; no causal ordering is established

Because dominant hand dexterity was associated with both aging and cognitive performance, mediation analyses were conducted to test whether dexterity statistically accounted for part of the age-related decline in cognition. A bootstrapped mediation analysis (5,000 iterations) examined whether dominant hand dexterity mediated the association between age and MoCA total score, with sex and race entered as covariates. Age was significantly associated with dexterity performance (path a: b = 0.080, standard error [SE] = 0.010, *p*<.001), indicating that older age was associated with slower peg completion times. Dexterity was in turn significantly associated with MoCA score (path b: b=-0.166, SE = 0.039, *p*<.001). The total effect of age on MoCA was significant (path c: b=-0.046, SE = 0.007, *p*<.001) and remained significant after accounting for dexterity (direct effect c’: b=-0.033, SE = 0.007, *p*<.001), consistent with partial statistical mediation. The indirect effect was statistically significant (a×b=-0.013, SEBOOT = 0.004, 95% CI [-0.022,-0.006]), with dexterity accounting for approximately 28.8% of the total age-related variance in MoCA performance. Because age, dexterity, and MoCA were measured concurrently, this quantity describes shared variance and does not establish that age acts on cognition through dexterity. This mediation model is illustrated in Fig. 7.

### Cross-sectional mediation: age, cerebellar Crus II volume, and grip strength

Given that age was associated with reduced grip strength and that Crus II volume correlated with grip performance, additional mediation analyses tested whether age-related reductions in cerebellar volume partially explained declines in grip strength. Parallel bootstrapped mediation analyses (5,000 iterations) tested whether Crus II gray matter volume mediated the relationship between age and grip strength, with sex and race as covariates. Older age was significantly associated with smaller Crus II volume (Crus II left, path *a*: *b*=-1.40 × 10^− 5^, SE = 1.52 × 10^− 6^, *p*<.001; Crus II right, path *a*: *b*=-1.19 × 10^− 5^, SE = 1.38 × 10^− 6^, *p*<.001). Larger Crus II volume was in turn significantly associated with stronger non-dominant grip strength (Crus II left, path *b*: partial *r*=.224, *p*<.001; Crus II right, path *b*: partial *r*=.198, *p*<.001).

Indirect effects through Crus II volume were significant for non-dominant grip: through Crus II left (-0.102, 95% CI [-0.157,-0.048]), accounting for approximately 41.4% of the total age effect on non-dominant grip; and through Crus II right (-0.085, 95% CI [-0.140,-0.037]), accounting for approximately 34.4%. Significant indirect effects were also observed for dominant grip through Crus II left (-0.064, 95% CI [-0.114,-0.017], 23.3% mediated) and Crus II right (-0.047, 95% CI [-0.095,-0.006], 16.8% mediated). In all models the total and direct associations of age with grip strength remained significant, consistent with partial statistical mediation rather than complete decomposition. Mediation results are summarized in Table 3. Of the two dominant-grip indirect effects, the one via right Crus II is best regarded as exploratory, because the corresponding partial correlation did not survive FDR correction (Table 2).

**Table 3:** Bootstrapped mediation of age effects on grip strength and cognition

Mediator	Outcome	Indirect effect	95% CI	% mediated
Crus II left	Grip non-dominant	−0.102	[− 0.157, − 0.048]	41.4%
Crus II right	Grip non-dominant	−0.085	[− 0.140, − 0.037]	34.4%
Crus II left	Grip dominant	−0.064	[− 0.114, − 0.017]	23.3%
Crus II right	Grip dominant	−0.047	[− 0.095, − 0.006]	16.8%
Dexterity (dom.)	MoCA total	−0.013	[− 0.022, − 0.006]	28.8%

Indirect effects, 95% percentile confidence intervals (5,000 bootstrap iterations), and proportion of the total age effect mediated, for each mediator → outcome pathway. Sex and race were entered as covariates in all paths. An indirect effect was considered significant when its 95% CI excluded zero

**Fig. 8:**
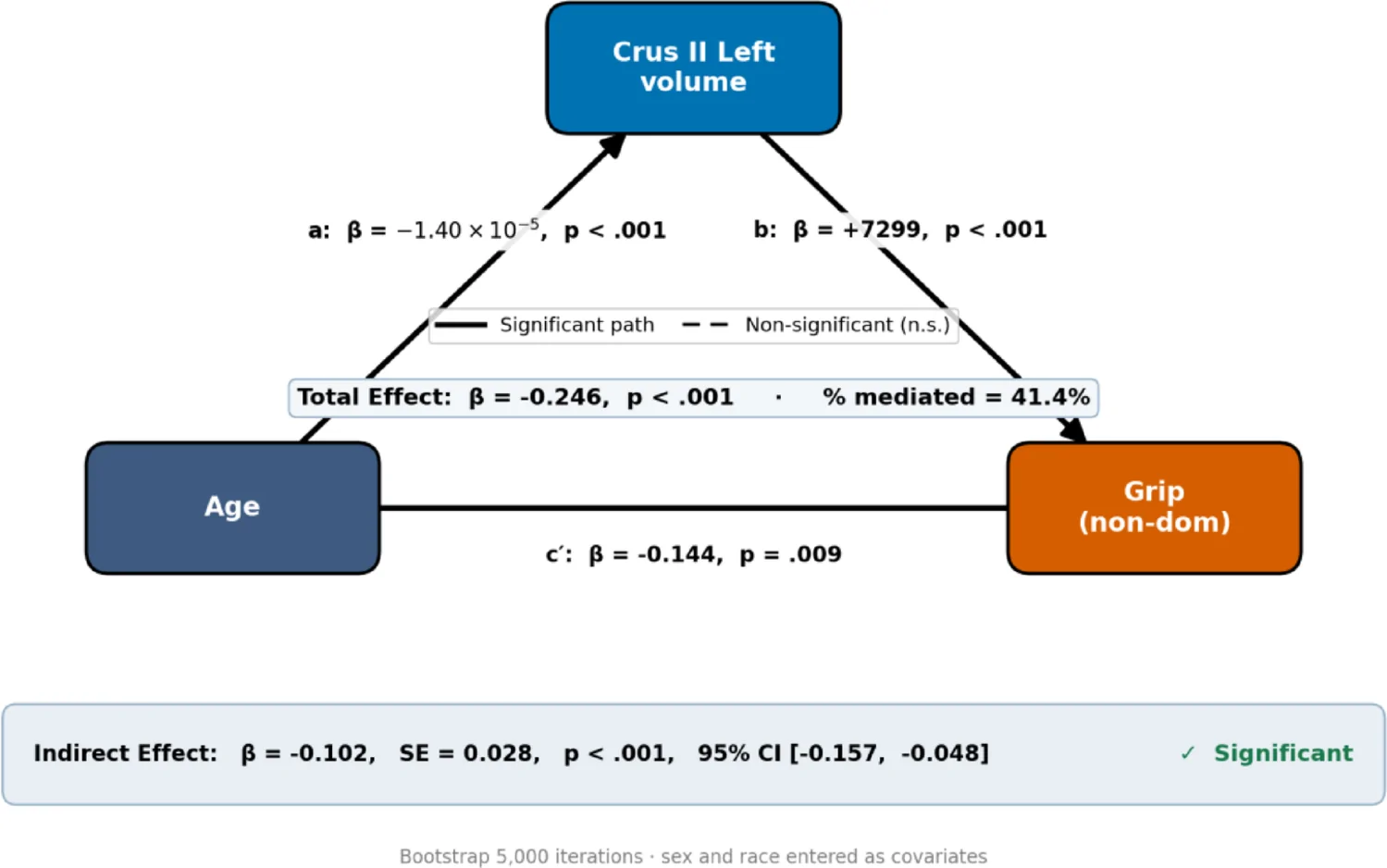
Cross-sectional mediation model relating age, left Crus II gray matter volume, and non-dominant grip strength. Mediation model with age as the independent variable, left Crus II volume as the mediator, and non-dominant grip strength as the outcome; sex and race entered as covariates. Path coefficients with standard errors and p-values are shown, together with the bootstrapped indirect effect (5,000 iterations, 95% CI) and the proportion of the total effect mediated. Solid arrows denote significant paths. Paths are estimated from cross-sectional data and describe a statistical decomposition of covariance; no causal ordering is established

The Crus II left → non-dominant grip mediation model is illustrated in Fig. 8.

### Formal test of the double dissociation

The hypothesized double dissociation was supported by direct comparison of the competing partial correlations (Table 4). Left Crus II volume was associated with non-dominant grip strength significantly more strongly than with dominant dexterity (Z = 2.33, *p* = .020) and with non-dominant dexterity (Z = 3.17, *p* = .002). Conversely, MoCA total score was associated with dominant dexterity significantly more strongly than with dominant grip strength (Z = 2.86, *p* = .004) and than with non-dominant grip strength (Z = 2.35, *p* = .019). The corresponding right Crus II contrast (grip versus dexterity) was in the same direction but did not reach significance (Z = 1.08, *p* = .280), consistent with the left-lateralized emphasis of the primary findings.

**Table 4:** Formal tests of the double dissociation (Meng et al. 1992 comparison of overlapping partial correlations)

Shared variable	Comparison (stronger vs. weaker)	*r* (stronger)	*r* (weaker)	Z	*p*
Left Crus II	grip (non-dom) vs. dexterity (dom)	0.223	0.046	2.33	0.020
Left Crus II	grip (non-dom) vs. dexterity (non-dom)	0.224	−0.016	3.17	0.002
MoCA total	dexterity (dom) vs. grip (dom)	0.240	0.035	2.86	0.004
MoCA total	dexterity (dom) vs. grip (non-dom)	0.227	0.049	2.35	0.019
Right Crus II	grip (non-dom) vs. dexterity (dom)	0.196	0.115	1.08	0.280

In each contrast the two partial correlations share a common variable (left or right Crus II volume, or MoCA total score). The r values are partial correlations controlling for age, sex, and race, with motor measures sign-aligned so that higher denotes better performance; Z and the two-tailed p-value test whether the two correlations differ. Rows 1–4 establish the dissociation for left Crus II and for cognition; the right Crus II contrast (row 5) is directional but non-significant. *n* = 296–297 per contrast. Correlations were computed on participants with complete data for all three variables in each contrast and may therefore differ slightly from those in Tables 2 and 5 and the results text

### Sensitivity analyses

#### Sex

Because female participants outnumbered male participants by roughly three to one, the two focal associations were re-estimated within each sex, controlling for age and race. The Crus II–grip association was present in both: for left Crus II with non-dominant grip, *r*=.245 (*p*=.040) in men (*n* = 74) and *r*=.218 (*p*=.001) in women (*n* = 224); for right Crus II, *r*=.253 (*p*=.033) and *r*=.174 (*p*=.010), respectively. Neither contrast differed significantly between sexes (Fisher’s Z = 0.21, *p*=.835 and Z = 0.61, *p*=.544). The dexterity–MoCA association was likewise present in both sexes and numerically stronger in men (*r*=-.356, *p*=.002) than in women (*r*=-.199, *p*=.003), again without a significant difference (Z=-1.25, *p*=.211). Restricting the analysis to the female subsample alone, the majority of the sex distribution in this study that hence carries the statistical power, left Crus II remained associated with non-dominant grip (*r*=.218, *p*=.001, *n* = 224), as did right Crus II (*r*=.174, *p*=.010). A formal Crus II × sex interaction term was nevertheless significant in the full sample for both left (standardized β=-0.178, *p*=.047) and right Crus II (β=-0.211, *p*=.020), indicating that the unstandardized slope relating Crus II volume to grip force is steeper in men. Higher mean and variance of grip force in men would produce a steeper unstandardized slope even under an identical underlying relation, and the standardized within-sex correlations, which are insensitive to that scaling, are closely similar. We note this as a plausible explanation rather than a demonstrated one. The principal association was therefore detectable in both sexes, but the significant interaction, together with a male subsample of 74, leaves the comparability of effect sizes across sexes unresolved; a sex-balanced replication is required to settle it.

#### Cognitive status and the MoCA ceiling

Spearman rank correlations of the covariate-adjusted residuals, which do not assume a linear relation between the adjusted variables, reproduced the dexterity–cognition findings: dominant dexterity with MoCA total score, ρ=-0.207 (*p*<.001), and with the memory index, ρ=-0.249 (*p*<.001; *n* = 298). Dichotomizing the sample at the conventional cut-off, 66 participants (21.7%) scored below 26 and 238 (78.3%) at or above it. The below-cut-off group was slower on the dominant-hand peg test (21.45 ± 4.42 s versus 18.62 ± 3.01 s; t = 4.87, *p*<.001, d = 0.75) but did not differ in grip strength (70.97 ± 21.08 lb versus 72.07 ± 23.64 lb; t=-0.36, *p*=.718, d=-0.05). In a logistic regression entering dexterity and grip strength simultaneously and controlling for age, sex, and race (*n* = 297; race collapsed to White versus non-White for this model, because the five-level coding produced quasi-complete separation within the 66-participant below-cut-off group), each additional second of dominant peg time was associated with 17.6% higher odds of scoring below the cut-off (odds ratio [OR] = 1.176, 95% CI [1.062, 1.302], *p*=.002), whereas grip strength was unrelated to cut-off status (OR = 1.005, 95% CI [0.985, 1.026], *p*=.615). The below-cut-off group also had smaller Crus II volumes bilaterally in unadjusted comparisons (left d=-0.45, *p*=.002; right d=-0.43, *p*=.004), but this difference was fully accounted for by the 10.8-year age difference between the groups and did not survive adjustment for age, sex, and race (left OR = 0.78, *p*=.417; right OR = 0.81, *p*=.520 per 0.001 proportion of total intracranial volume). The dissociation thus holds on a clinically dichotomized outcome that is less sensitive to compression in the upper tail, though dichotomization does not remove measurement error or the general limitations of a screening instrument.

#### Age stratum

Splitting the sample at 50 years, the Crus II–grip association was present in both younger adults (20–49; left Crus II *r*=.228, *p*=.006; right *r*=.199, *p*=.017; *n* = 148) and older adults (50–79; left *r*=.167, *p*=.046; right *r*=.183, *p*=.028; *n* = 150), with no significant difference between strata (Fisher’s Z ≤ 0.54, *p*≥.587). The dexterity–cognition association was carried predominantly by the older stratum (MoCA total *r*=-.249, *p*=.002; memory index *r*=-.206, *p*=.013; *n* = 152) and was non-significant among younger adults (*r*=-.130, *p*=.125 and *r*=-.091, *p*=.283; *n* = 146), although the two strata did not differ significantly (Z = 1.06, *p*=.291 and Z = 1.00, *p*=.316). This is the pattern expected if fine motor performance indexes cognitive aging rather than stable individual differences, but in the absence of significant moderation it should be treated as suggestive. Sensitivity results are summarized in Table 5.

**Table 5:** Sensitivity analyses by sex, age stratum, and cognitive status

Analysis	Stratum / model	Estimate	*p*	*n*
**Sex-stratified analyses (controlling for age and race)**				
Crus II L × grip non-dom	Men	*r* = .245	0.040	74
	Women	*r* = .218	0.001	224
	Fisher’s Z (men vs. women)	Z = 0.21	0.835	n/a
Crus II R × grip non-dom	Men	*r* = .253	0.033	74
	Women	*r* = .174	0.010	224
	Fisher’s Z (men vs. women)	Z = 0.61	0.544	n/a
Dexterity dom × MoCA total	Men	*r* = −.356	0.002	74
	Women	*r* = −.199	0.003	224
	Fisher’s Z (men vs. women)	Z = −1.25	0.211	n/a
**Crus II × sex interaction (full sample)**				
Crus II × sex interaction	Left, predicting grip non-dom	β = −0.178	0.047	298
	Right, predicting grip non-dom	β = −0.211	0.020	298
**Age-stratified analyses (controlling for age, sex, and race)**				
Crus II L × grip non-dom	Younger (20–49)	*r* = .228	0.006	148
	Older (50–79)	*r* = .167	0.046	150
	Fisher’s Z (younger vs. older)	Z = 0.54	0.587	n/a
Crus II R × grip non-dom	Younger (20–49)	*r* = .199	0.017	148
	Older (50–79)	*r* = .183	0.028	150
	Fisher’s Z (younger vs. older)	Z = 0.13	0.894	n/a
Dexterity dom × MoCA total	Younger (20–49)	*r* = −.130	0.125	146
	Older (50–79)	*r* = −.249	0.002	152
	Fisher’s Z (younger vs. older)	Z = 1.06	0.291	n/a
Dexterity dom × MoCA memory index	Younger (20–49)	*r* = −.091	0.283	146
	Older (50–79)	*r* = −.206	0.013	152
	Fisher’s Z (younger vs. older)	Z = 1.00	0.316	n/a
**Cognitive status / MoCA ceiling (full sample)**				
Rank-based sensitivity	Dexterity dom × MoCA total	ρ = −0.207	< 0.001	298
	Dexterity dom × memory index	ρ = −0.249	< 0.001	298
MoCA < 26 (logistic)	Dominant dexterity (per second)	OR = 1.176 [1.062, 1.302]	0.002	297
	Dominant grip strength (per lb)	OR = 1.005 [0.985, 1.026]	0.615	297

Partial correlations within each sex stratum control for age and race; partial correlations within each age stratum control for age, sex, and race. Significance is evaluated on *n* − 2 - k degrees of freedom, where k is the number of covariates. Fisher’s Z tests whether the two stratum-specific correlations differ. The Crus II × sex interaction is estimated in the full sample with all continuous variables standardized. The logistic regression predicts MoCA below cut-off (< 26) from dominant dexterity and dominant grip strength entered simultaneously, controlling for age, sex, and race. Spearman values are rank correlations of the covariate-adjusted residuals. These analyses were not FDR-corrected and are reported as sensitivity checks

## Discussion

The present study examined associations between cerebellar Crus I/II gray matter volume, grip strength, manual dexterity, and cognitive performance in 304 healthy adults, controlling for age, sex, and race. Three primary findings emerged. First, bilateral Crus II volume was robustly associated with grip strength and statistically accounted for a substantial share of the age-related variance in grip. Second, neither Crus I nor Crus II volume was significantly associated with dexterity, despite adequate power to detect moderate effects. Third, dominant hand dexterity was selectively associated with MoCA total score and the memory index and statistically accounted for a comparable share of the age-related variance in cognition. Grip strength and dexterity, though related, thus have dissociable cerebellar and cognitive correlates: posterior cerebellar volume is associated with grip capacity, whereas dexterity is associated with cognitive status independently of Crus I/II volume.

The finding that bilateral Crus II volume correlates positively with grip strength, surviving FDR correction with partial correlations ranging from *r*=.156 to *r*=.224, represents the primary structural finding of this study. In cross-sectional mediation models, Crus II volume statistically accounted for 34.4–41.4% of the age-related variance in non-dominant grip strength; because all variables were measured concurrently, this quantifies shared variance rather than a demonstrated causal chain. These results align with converging evidence implicating Crus I and Crus II in higher-order functions and identifying posterior cerebellar atrophy as a sensitive indicator of neural aging (Ramanoël et al. 2018; Van Rossem et al. 2025). The present data extend these observations to grip strength, identifying Crus II as a cerebellar marker of grip capacity. Convergent evidence comes from infratentorial vascular burden: in an independent sample of 1,725 stroke patients, infratentorial cerebral microbleed count was positively associated with the brain age gap across nine sensorimotor regions, indicating that posterior fossa microvascular injury tracks accelerated aging in functionally connected motor cortices (Pallapothu et al. 2026). The present results sit comfortably within that network-level framing, in that a posterior cerebellar volume tracks a motor phenotype expressed through those same cortical systems.

The grip–Crus II association was most consistent for non-dominant grip across both hemispheres. One interpretation is that non-dominant grip, being less influenced by task-specific practice and habitual hand use, may more directly index central cerebellar integrity than dominant grip, which is more strongly shaped by peripheral neuromuscular factors and lateralized skill. This interpretation is speculative and warrants longitudinal or intervention-based study. Crus I showed numerical trends in the same direction as Crus II that did not survive FDR correction, consistent with the two regions occupying adjacent positions within the cognitive cerebellum but possibly reflecting greater statistical sensitivity for Crus II in the present sample.

The Crus II–grip association was not paralleled by a Crus II–dexterity association. No Crus I or Crus II region showed a significant relationship with Nine-Hole Peg Test performance after FDR correction, with the largest uncorrected effect being *r*=-.10. The present study was powered to detect correlations of *r*≥.16 at 80%, meaning that moderate-to-large effects of Crus I/II on dexterity can be excluded with reasonable confidence, though small effects below this threshold cannot be ruled out. This dissociation suggests that Crus II volume is specifically associated with the motor capacity component captured by grip strength rather than with the fine coordinated movement captured by dexterity.

This dissociation was statistically reliable, not an artifact of comparing a significant with a non-significant correlation: left Crus II volume was associated with grip significantly more strongly than with dexterity, and MoCA with dexterity significantly more strongly than with grip (all *p* < .05; Table 4). Its direction may initially seem counterintuitive, because Crus I/II are usually framed as cognitive regions and dexterity is the more cognitively demanding task. Two considerations help reconcile this. First, Crus II volume is a slowly accruing structural index of neural integrity, whereas Nine-Hole Peg Test performance is state-sensitive, shaped by attentional, peripheral, and practice factors; volume may therefore track the stable, capacity-like component that grip also reflects, rather than the dynamic online-control demands of pegboard performance. Second, the grip–Crus II association need not imply that Crus II executes gross movement: posterior cerebellar volume covaries with global brain and neuromuscular aging, so it may index a whole-organism integrity expressed more faithfully in maximal force output than in a brief, ceiling-prone dexterity task. Dexterity thus carries cognitive information that is not localized to Crus I/II volume, as evidenced by its selective link to the MoCA memory index, whereas Crus II volume indexes a strength-relevant integrity that is cognitively non-specific. These interpretations are post hoc and should be tested with task-based functional imaging and longitudinal designs.

Dominant hand dexterity was selectively associated with MoCA total score and the memory index following FDR correction, with no other subscores surviving. These findings are consistent with evidence that dexterity-based measures are sensitive, independent predictors of cognition in older adults (Schneider et al. 2025), and extend that work by demonstrating selectivity for the memory domain, namely delayed recall and recognition, which are among the domains most sensitive to early neurodegenerative change. Grip strength was not significantly associated with any MoCA subscore, consistent with the hypothesis that dexterity, rather than gross strength, is the more sensitive motor correlate of cognitive status.

In the corresponding cross-sectional mediation model, dexterity statistically accounted for 28.8% of the association between age and MoCA total score. The direct effect remained significant after dexterity was entered, so the decomposition is partial rather than complete, consistent with age-related differences in cognition being associated with several measured and unmeasured factors, of which fine motor performance is one. We emphasize again that this model cannot adjudicate direction: slowing dexterity may index the same neural changes that produce cognitive decline, cognitive decline may degrade performance on a timed manual task, or both may follow from a shared upstream process. The value of the estimate is in establishing that the shared variance is substantial and that it is specific to dexterity rather than to motor function generally, not in establishing a mechanism.

Two features of the cognitive measure temper this conclusion and, in one respect, strengthen it. The MoCA in this healthy sample was severely restricted in range: nearly one participant in five scored the maximum of 30, more than half scored within two points of it, and the memory index was at ceiling for over half the sample. Range restriction of this kind biases correlations toward zero, so the dexterity–cognition associations reported here are conservative estimates, and the null result for grip strength carries a correspondingly weaker evidential burden: a genuinely small grip–cognition association could have been suppressed below detectability. Against that, the dissociation did not depend on the distributional properties of the MoCA. It reproduced under Spearman rank correlation of the covariate-adjusted residuals, which does not assume a linear relation between the adjusted variables, and it reproduced when the sample was dichotomized at the clinical cut-off of 26, where each additional second of dominant peg time carried roughly 18% higher odds of scoring below cut-off while grip strength carried none. That a clinically framed outcome, less sensitive to upper-tail compression, recovers the same asymmetry is the strongest evidence available to us that the dissociation reflects the motor measures rather than the scaling of the cognitive instrument. It does not remove measurement error, nor the broader limitations of a screening test. A battery with greater ceiling headroom in healthy adults, for example an episodic memory composite or a processing speed measure, remains the appropriate next test, and would also permit a fairer test of the grip strength null.

The sex composition of the cohort (229 women, 75 men) is a genuine constraint on the grip strength findings in particular, because grip strength is the measure in this study with by far the largest sex difference. Three checks indicate that the Crus II–grip association is not attributable solely to that imbalance. The association was significant within each sex analyzed separately, with near-identical partial correlations (men *r*=.245, women *r*=.218 for left Crus II); it remained significant in the female-only subsample, which carries the bulk of the statistical power; and Fisher’s r-to-z provided no evidence of a sex difference in either hemisphere. A formal interaction term was nonetheless significant, indicating a steeper unstandardized slope in men. The larger mean and variance of male grip force would produce this even under an identical underlying relation, and the standardized within-sex correlations are consistent with that account, but we cannot regard it as demonstrated: sex moderation cannot be excluded on the present data. What the imbalance does limit is precision: the male estimates rest on 74 participants, moderation tests at that cell size are underpowered against small true differences, and the generalizability of the effect magnitude to men cannot be regarded as established. A sex-balanced replication is the clearest single improvement a future study could make to this line of work.

### Atlas choice and cerebellar parcellation

The present analyses used the Automated Anatomical Labeling 3 (AAL3) atlas (Rolls et al. 2020; Tzourio-Mazoyer et al. 2002) for cerebellar parcellation, as bundled within the CAT12 VBM pipeline (Gaser et al. 2024). We acknowledge that the Spatially Unbiased Infratentorial Template (SUIT) cerebellar atlas (Diedrichsen et al. 2009) provides higher-resolution lobular boundaries and is widely used in cerebellum-focused research. AAL3 was selected for three reasons. First, its Crus I and Crus II are anatomically defined whole-lobule units whose boundaries correspond well to the same-named SUIT lobules. Second, our a priori hypotheses concerned whole-lobule volumes rather than subregional gradients. Third, the AAL family is among the most widely deployed atlases in neuroaging and dementia VBM, affording direct comparability with prior cerebellar studies. We regard AAL3 as a conservative choice prioritizing comparability and reproducibility; replication in SUIT-parcellated data is recommended.

### Limitations and future studies

Several limitations must be acknowledged. First, the cross-sectional design precludes causal inference. All associations reported here, including every path in the mediation models, are correlational; the mediation results describe how concurrent variance is partitioned and do not establish that age acts on grip strength through cerebellar atrophy, or on cognition through dexterity. Second, MoCA total scores exhibited a pronounced ceiling in this cognitively healthy sample (19.4% at maximum; skewness − 1.27), which attenuates cognitive associations and limits the evidential weight of the grip strength null; the rank-based and dichotomized analyses reported above mitigate but do not eliminate this concern. Third, the sample comprised substantially more female (*n* = 229) than male (*n* = 75) participants. Although the Crus II–grip association held within each sex and in the female subsample alone, the male estimates are imprecise and the moderation tests are underpowered, so the magnitude of the effect in men should be regarded as provisional. Fourth, the sample was drawn from a single academic medical center in the southeastern United States and was predominantly White (82.6%), limiting demographic and geographic generalizability. Fifth, while the study was adequately powered to detect moderate effects (*r*≥.16), small true associations between Crus I/II volume and dexterity cannot be excluded. Sixth, gray matter volume is a coarse structural index that does not distinguish among neuronal loss, dendritic simplification, and glial change, and the AAL3 whole-lobule parcellation cannot resolve subregional gradients within Crus I/II. Finally, all data were acquired at a single time point, precluding assessment of trajectory-based associations; a longitudinal follow-up of this cohort is the natural test of the hypotheses generated here.

## Conclusion

This study provides cross-sectional evidence for a double dissociation in the cerebellar and cognitive correlates of grip strength and manual dexterity. Cerebellar Crus II volume is robustly and bilaterally associated with grip strength, surviving FDR correction, and statistically accounts for a substantial share of the age-related variance in grip. In contrast, manual dexterity is selectively linked to MoCA total score and the memory index, also FDR-robust, and statistically accounts for a comparable share of the age-related variance in cognition, while showing no significant structural association with cerebellar Crus I/II. The Crus II–grip association was present within each sex and within both age strata; the dexterity–cognition association was present within each sex and in the older stratum, with between-stratum differences not reaching significance. Both should be understood as concurrent associations awaiting longitudinal test. These findings underscore the importance of differentiating motor measures in the study of cerebellum–behavior relationships in aging: grip strength and dexterity are related motor constructs with distinct cerebellar and cognitive signatures, and treating them as interchangeable risks obscuring both the cerebellar structural correlates of strength and the cognitive relevance of fine motor coordination.

## Supplementary Information

Below is the link to the electronic supplementary material.

Supplementary Material 1

## Data Availability

The data that support the findings of this study were obtained from the University of South Carolina Aging Brain Cohort (ABC) Study Repository. These data are owned and maintained by the ABC study, and are not publicly available, as they contain information that could compromise the privacy of individual participants. De-identified data are available from the University of South Carolina Aging Brain Cohort Study Repository on reasonable request, with the permission of the ABC study team and subject to a data use agreement and the relevant institutional and ethical (IRB) approvals.

## Code availability

All analysis code is provided with this article as the archive dex_grip_analysis_code.zip, which reproduces every value reported in Tables 1, 2, 3, 4 and 5 and in the Sensitivity Analyses once the dataset has been obtained and placed as described in the archive. The code contains no participant-level data.
