# Supplementary material for: Quantifying dexterity and grip strength associations between age, cognitive function (MoCA), and cerebellar gray matter volume in healthy adults across the adult lifespan
Source: Brain Struct Funct. 2026 Oct 22;231(8):156. doi: 10.1007/s00429-026-03213-6 (PMC13690861; doi:10.1007/s00429-026-03213-6)
Supplement: Supplementary file 1 [file 429_2026_3213_MOESM1_ESM.docx]

**Online Resource 1**

Supplementary material for: Quantifying Dexterity and Grip Strength Associations between Age, Cognitive Function (MoCA), and Cerebellar Gray Matter Volume in Healthy Adults across the Adult Lifespan.

**Contents**

Fig. S1 Distributions of grip strength, dexterity, and cognition by race

Fig. S2 Behavioral topography across the 26 cerebellar parcels

**Fig. S1** Distributions of grip strength, dexterity, and cognition by race. Box-and-jitter plots of dominant grip strength (lb), dominant Nine-Hole Peg Test time (s), and MoCA total score across racial groups (Asian, Black/African American, White, Unknown). One-way analysis of variance (ANOVA) F statistics, p-values, and group sizes are inset. Race was dummy-coded and entered as a covariate in every inferential model reported in the main text, and gray matter volume is not reported by race; these panels are therefore provided for descriptive completeness only and support no inferential comparison. The single participant reporting more than one race is omitted from these panels. N = 304.

**Fig. S2** Behavioral topography across the 26 cerebellar parcels. (a) Each of the 26 cerebellar parcels of the AAL3 atlas, colored by its partial correlation with the named measure after partialling age, sex, and race, in posterior and inferior views. Outlined parcels survive Benjamini–Hochberg FDR correction across the 26 parcels within that measure. Positive r indicates that larger volume accompanies a higher score on that measure; higher 9HPT time denotes slower performance. (b) The same partial correlations shown as a grid, with the parcels ordered by cerebellar subdivision. Values are printed for the four a priori regions of interest, which are outlined; an asterisk marks survival of FDR correction across the 26 parcels, and an open circle marks an uncorrected p<.05. These analyses are post hoc and exploratory, and the FDR family used here is separate from the pre-specified families reported in the main text (see Statistical Methods)
